# P-219. A Comparison of Clinical Outcomes for Polymerase Chain Reaction-positive (PCR) Enzyme Immunoassay-negative (EIA) Patients to PCR+/ EIA + Patients Diagnosed with *Clostridioides difficile*

**DOI:** 10.1093/ofid/ofae631.423

**Published:** 2025-01-29

**Authors:** Philip Jurasinski, Greeshma Erasani, Xiangni Wu, Yazan Sallam, Noor Hassan, Nicholas Bennett, Sarah E Boyd

**Affiliations:** University of Missouri-Kansas City, Kansas City, Missouri; University of Missouri-Kansas City, Kansas City, Missouri; University of Missouri-Kansas City, Kansas City, Missouri; University of Missouri-Kansas City, Kansas City, Missouri; University of Missouri-Kansas City, Kansas City, Missouri; Saint Luke's Health System, Kansas City, Missouri; Saint Luke's Health System, Kansas City, Missouri

## Abstract

**Background:**

In August 2021, Saint Luke’s Health System transitioned *Clostridioides difficile (C. diff.)* testing from polymerase chain reaction (PCR) only to two-step enzyme immunoassay (EIA) reflex following PCR+ for suspected *C. diff*. infection. Uncertainty in management may arise when PCR and EIA test results differ. Previous studies suggested that disease severity varies when results return as PCR+/EIA- due to possible colonization. Clinicians may treat patients if there is no other explanation for diarrhea, clinical instability, previous antibiotic use, immunocompromised status, or if recommended by infectious diseases/gastroenterology consultants. We compared clinical outcomes of patients with PCR+/EIA+ to those with PCR+/EIA- results.

**Table 1:**
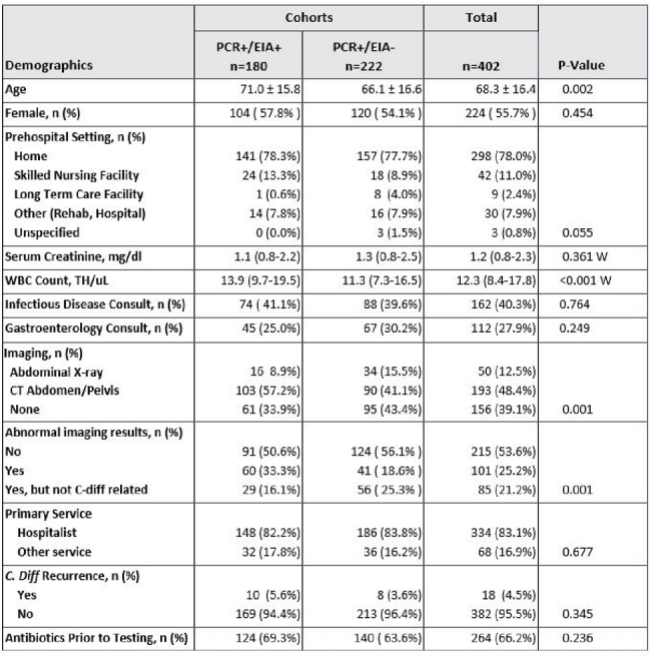
Patient Demographics Continuous variables compared using Student's T-test. Categorical variables compared using chi-square or Fisher's exact test. W = Wilcoxon rank-sum test.

**Methods:**

This was a retrospective cohort study from August 2021 to August 2023 in a multi-site, integrated health system, comparing patients with PCR+/EIA- *C. diff*. test results to those with PCR+/EIA+ results. Patients were included if they were 18 years or older and needed *C diff* testing. Patients with incomplete data were excluded. We defined full treatment as 10 or more days of *C. diff*. treatment, partial as 2 to 9 days and no treatment as 2 or fewer days. The primary outcome was length of stay. Secondary outcomes were readmission rates, colectomy rates, intensive care unit (ICU) admission, and the percentage of patients with resolved diarrhea on day of discharge. An additional subgroup analysis compared fully treated PCR+/EIA+ patients to patients with partially or untreated PCR+/EIA- results.

**Table 2:**
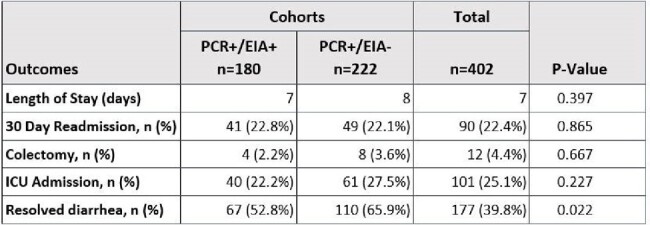
Outcomes in PCR + / EIA- vs. PCR +/ EIA + Groups Continuous variables compared using Student's T-test. Categorical variables compared using chi-square or Fisher's exact test. W = Wilcoxon rank-sum test.

**Results:**

222 patients had PCR+/EIA- and 180 patients had PCR+/EIA+ results. For the primary outcome, no difference was detected between cohorts including the subgroup analysis. Diarrhea resolution occurred less often in the PCR+/EIA- group, including the subgroup analysis. See Tables 1-4 for more information.

**Table 3:**
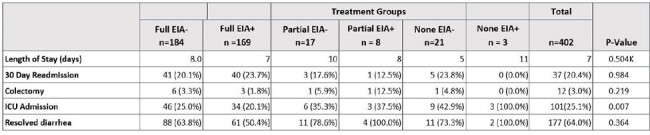
Outcomes in PCR+/EIA- vs. PCR+/EIA+ Groups Analyzed by Treatment Groups Continuous variables compared using one-way analysis of variance. Categorical variables compared using chi-square or Fisher's exact test. K = Kruskal-Wallis test.

**Conclusion:**

This study found no difference in length of stay, but lower diarrhea resolution in PCR+/EIA- patients. Notably, high treatment rates occurred in both cohorts, limiting generalizability. More research should be conducted to help clinicians discern treatment of discordant two-step test results, particularly in settings with lower treatment rates of PCR+/EIA- results.

**Table 4:**
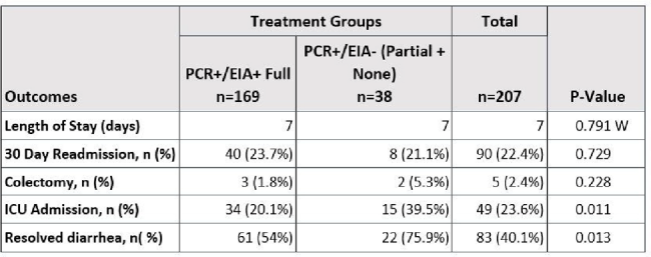
Outcomes in PCR+/EIA+ Full Treatment Group vs. PCR+/EIA- (Partial + No Treatment) Group Continuous variables compared using Student's T-test. Categorical variables compared using chi-square or Fisher's exact test. W = Wilcoxon rank-sum test.

**Disclosures:**

**All Authors**: No reported disclosures

